# Composite immune marker scores associated with severe mental disorders and illness course

**DOI:** 10.1016/j.bbih.2022.100483

**Published:** 2022-07-02

**Authors:** Monica Bettina Elkjaer Greenwood Ormerod, Thor Ueland, Maren Caroline Frogner Werner, Gabriela Hjell, Linn Rødevand, Linn Sofie Sæther, Synve Hoffart Lunding, Ingrid Torp Johansen, Torill Ueland, Trine Vik Lagerberg, Ingrid Melle, Srdjan Djurovic, Ole Andreas Andreassen, Nils Eiel Steen

**Affiliations:** a NORMENT Centre, Division of Mental Health and Addiction, Oslo University Hospital, Oslo, Norway; b Institute of Clinical Medicine, University of Oslo, Oslo, Norway; c Research Institute of Internal Medicine, Oslo University Hospital, Rikshospitalet, Oslo, Norway; d KG Jebsen Inflammatory Research Center, University of Oslo, Oslo, Norway; e Department of Psychiatry, Østfold Hospital, Graalum, Norway; f Department of Psychology, University of Oslo, Oslo, Norway; g Department of Medical Genetics, Oslo University Hospital and University of Oslo, Oslo, Norway; h NORMENT, Department of Clinical Science, University of Bergen, Bergen, Norway

**Keywords:** Principal component analysis, Composite scores, Severe mental disorders, Schizophrenia, Bipolar disorder, Immune marker, Neuroinflammation, PCA, Principal Component Analysis, SMD, Severe Mental Disorders, SCZ, Schizophrenia, BD, Bipolar Disorder

## Abstract

**Background:**

Low-grade inflammation has been implicated in the pathophysiology of severe mental disorders (SMDs) and a link between immune activation and clinical characteristics is suggested. However, few studies have investigated how patterns across immune markers are related to diagnosis and illness course.

**Methods:**

A total of 948 participants with a diagnosis of schizophrenia (SCZ, N = 602) or bipolar (BD, N = 346) spectrum disorder, and 814 healthy controls (HC) were included. Twenty-five immune markers comprising cell adhesion molecules (CAMs), interleukin (IL)-18-system factors, defensins, chemokines and other markers, related to neuroinflammation, blood-brain barrier (BBB) function, inflammasome activation and immune cell orchestration were analyzed. Eight immune principal component (PC) scores were constructed by PC Analysis (PCA) and applied in general linear models with diagnosis and illness course characteristics.

**Results:**

Three PC scores were significantly associated with a SCZ and/or BD diagnosis (HC reference), with largest, however small, effect sizes of scores based on CAMs, BBB markers and defensins (p < 0.001, partial η^2^ = 0.02–0.03). Number of psychotic episodes per year in SCZ was associated with a PC score based on IL-18 system markers and the potential neuroprotective cytokine A proliferation-inducing ligand (p = 0.006, partial η^2^ = 0.071).

**Conclusion:**

Analyses of composite immune markers scores identified specific patterns suggesting CAMs-mediated BBB dysregulation pathways associated with SMDs and interrelated pro-inflammatory and neuronal integrity processes associated with severity of illness course. This suggests a complex pattern of immune pathways involved in SMDs and SCZ illness course.

## Highlights

• Composite score of VCAM-1, ICAM-1, NCAD and IL-18BP associated with SCZ and BD.
• Composite score of MadCAM-1 and BD-1 associated with SCZ and BD.
• Composite score of S100B, furin, HNP1-3 and BD-1 associated with BD.
• Composite score of APRIL and IL-18R markers associated with psychotic episode rate.

## Graphical abstract

**Figure:**
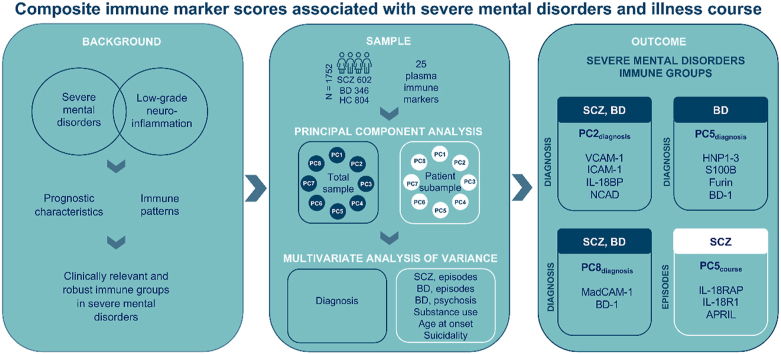

## 1 Introduction

Schizophrenia and bipolar disorder are severe mental disorders (SMDs) with shared clinical characteristics and genetic underpinnings (McCutcheon et al., 2020; Purcell et al., 2009). SMDs are associated with considerable disability and suffering; however, disease course varies between individuals (Charlson et al., 2018; Ferrari et al., 2016). Up to one third of patients with schizophrenia experience a chronic course with limited effect of therapy and a similar proportion has a benign course (Marder and Cannon, 2019). Likewise, the course of bipolar disorder varies significantly between afflicted individuals (Lagerberg et al., 2021; Turvey et al., 1999). The heterogeneity probably reflects different underlying disease mechanisms.

There are several lines of evidence of immune system involvement in the pathophysiology of SMDs (Miller and Goldsmith, 2017; Munkholm et al., 2013). Large registry studies and meta-analyses have recently shown a strong relationship of SMDs with infections and autoimmune disorders (Benros et al., 2011; Bergink et al., 2014; Cullen et al., 2019; Köhler-Forsberg et al., 2019; Najjar et al., 2018). Genome-wide association studies (GWAS) link the immune-related major histocompatibility complex (MHC) locus with both schizophrenia (Ripke et al., 2014) and bipolar disorder (Mullins et al., 2021). Also, immune genetic associations are found outside the MHC region and there is genetic overlap with immune mediated disorders (Pouget, 2018). Imaging techniques have identified neuroinflammation (Pasternak et al., 2016), in line with findings of elevated microglia cell activity in studies of postmortem brain tissue (Trepanier et al., 2016) and immune marker aberrations in cerebrospinal fluid (Bechter et al., 2010). Nevertheless, knowledge about low-grade inflammation in SMDs is mainly based on reports of altered circulating immune marker levels (Benedetti et al., 2020; Frydecka et al., 2018; Goldsmith et al., 2016; Khoury and Nasrallah, 2018). Such immune markers, constituting an extensive number of cytokines and adhesion molecules (Kroken et al., 2018), are involved in complex interacting immune regulatory mechanisms, potentially related to the increased cardiovascular disease risk in SMDs (De Hert et al., 2018; Reponen et al., 2020). Determinants of immune marker levels are mainly unknown, although a major impact of non-heritable factors is indicated (Brodin et al., 2015), in particular acquired metabolic disturbances (Huet et al., 2021; Makki et al., 2013), and interactions with endocrine systems (Taub, 2008). However, as studies of systemic immune abnormalities in SMDs are based on individual markers (Dickerson et al., 2016; Kroken et al., 2018), common underlying mechanisms have been challenging to uncover, and findings are prone to spurious variation.

A recent systematic review of meta-analyses suggested diagnosis-related as well as state specific immune marker abberration in SMDs (Yuan et al., 2019). However, studies of clinical state showed significant inconsistencies. In general, studies of symptom severity and immune markers are susceptible to interfering effects of stress (Lataster et al., 2013; Segerstrom and Miller, 2004; Tourjman et al., 2013). Only a few studies report associations between immune markers and illness course characteristics (De Picker et al., 2017; Lizano et al., 2020), such as suicidality (Black and Miller, 2015; Isung et al., 2020). To improve clinical relevance and interpretability of SMDs - immune associations, we propose to analyze distinct groups in terms of illness course characteristics not dependent on current clinical state. Severity of illness course is often described quantitatively by number of illness episodes (Coulon et al., 2020; Immonen et al., 2017; Kennedy et al., 2015) together with qualitative markers of comorbid substance use disorder (Kendler et al., 2019; Messer et al., 2017), history of suicidality (Yates et al., 2019), and in bipolar disorders, presence of psychotic episodes (Burton et al., 2018; Keck et al., 2003). A more severe illness course also seems to be related to an earlier age at onset (Cirone et al., 2021; Coulon et al., 2020; Hanssen et al., 2015). To address the complex patterns of the immune system, the current study uses Principal Component Analysis (PCA) of immune markers. By obtaining composite immune scores in an exploratory approach, more of the interplay of the immune pathways underlying the phenotypes relative to single immune marker analyses, might be indicated (Miller and Goldsmith, 2017).

We aim to identify immune marker components associated with diagnosis and illness course characteristics. To be able to indicate the potential small effects of immune components in the complex mechanisms underlying these phenotypes, we apply a large sample of SMDs and healthy controls (HC). Associations of immune markers with complex illness course characteristics are anticipated to be small in these highly complex and multifaceted mechanisms, however the potential immune components might prove meaningful in the long term. On the basis of the inflammatory model of SMDs, a set of generally stable and abundantly expressed immune markers encompassing novel markers and markers with established link to SMDs, representing pathways of potential pathophysiological relevance, including neuroinflammation, blood-brain barrier (BBB) function, inflammasome activation and immune cell orchestration, were chosen. Commonly used illness course characteristics were analyzed, including number of illness episodes (Häfner, 2019; Luciano et al., 2021; Peters et al., 2016; Provenzani et al., 2021), suicide attempts (Black and Miller, 2015; Yates et al., 2019), comorbid substance use disorder (Kendler et al., 2019; Messer et al., 2017) and for bipolar disorder presence of psychotic features (Burton et al., 2018; Keck et al., 2003).

## 2 Methods

### 2.1 Study setting

Participants were included through the Thematically Organized Psychosis (TOP)-study at the Norwegian Center for Mental Disorder Research (NORMENT). Recruitment of patients is conducted from the major hospitals in Oslo, currently covering a catchment area of approximately 700,000 inhabitants. Patient inclusion criteria are age range from 18 to 65 years and meeting the Diagnostic Manual of Mental Disorders (DSM)-IV (First, 2013) criteria for schizophrenia spectrum or bipolar spectrum disorders. Exclusion criteria are IQ < 70, severe somatic illness, neurological disorder, or a history of moderate or severe head trauma. HC were randomly selected from the same catchment area and age range, using statistical records. HC were excluded based on the same criteria in addition to symptoms of SMDs, current substance abuse, and history of SMDs in close relatives. Furthermore, only individuals with sufficient Scandinavian language skills to complete the assessments were included. All participants gave informed written consent. For the current study, participants with CRP above 10.0 mg/L were excluded to prevent acute infections influencing the immune markers (Povoa, 2002).

### 2.2 Sample

Participants of the TOP sample with immune assessments (N = 1762) consisted of N = 602 patients with schizophrenia spectrum disorders (SCZ), here including schizophrenia (N = 345), schizophreniform disorder (N = 36), schizoaffective disorder (N = 86), delusional disorder (N = 40), brief psychotic disorder (N = 8), and psychotic disorder not otherwise specified (NOS, N = 87), N = 346 patients with bipolar spectrum disorders (BD), here including bipolar I disorder (N = 216), bipolar II disorder (N = 112) and bipolar disorder NOS (N = 18), and N = 814 HC. Immune data overlapping with the current study have been published elsewhere (Andreou et al., 2021; Engh et al., 2021; Sheikh et al., 2022; Szabo et al., 2022).

### 2.3 Clinical assessments

Trained psychologists and physicians conducted interviews of the patient participants. Sociodemographic and medical history were collected, and diagnostic interviews were performed using the Structured Clinical Interview for DSM-IV Axis I Disorders (SCID-1) (Spitzer et al., 1992), including substance use disorders and number of psychotic and affective episodes. The clinical interviewers participated in regular diagnostic meetings and were supervised by senior researchers. Inter-rater reliability of the diagnostics was good, with an overall kappa score of 0.77 (95% CI: 0.60–0.94) (Ringen et al., 2008). Current symptom levels were assessed with Global Assessment of Functioning (GAF) – symptoms scale (Pedersen and Karterud, 2012) and The Positive and Negative Syndrome Scale (PANSS) (Kay et al., 1987). Routine blood tests and a physical examination including height and weight for body mass index (BMI) was performed in all participants and within two weeks of symptom assessments for patients. Information about psychopharmacological treatment was obtained from interviews and medical records, and categorized into use of antipsychotics (yes/no), antidepressants (yes/no), and anticonvulsants and/or lithium (yes/no). Details of anti-inflammatory drug use in patients were similarly recorded and is given in Supplementary Table 1.

### 2.4 Inflammatory markers

Blood samples were drawn from the antecubital vein on EDTA vials and plasma stored at −80 °C for later immunological analyses. The patient subsample had blood withdrawn earlier in the day (average at 10 a.m.) than HC (average at 3 p.m.). The samples were stored on average 5–8 years (range 3–10), with shorter duration in HC. Freezer storage time was controlled for in the analyses. Twenty-five inflammatory markers were analyzed at the Research Institute of Internal Medicine, Oslo University Hospital. Applying enzyme immunoassays (EIA), samples were analyzed in duplicate by use of antibodies available from R&D systems (Minneapolis, MN, USA) in a 384 format, using a combination of a pipetting robot from Selma and a dispenser/washer from Biotek. We used an ELISA plate reader (BIO-RAD, Hercules, CA, USA) read the absorbance at 450 nm with wavelength correction set 540 nm. In all EIAs, intra- and inter-assay coefficients of variation were <10%. See Supplementary Table 5 for immunoassay details and characteristics.

We analyzed a broad range of immune markers with a potential link to SMDs: chemokines, cell adhesion molecules (CAMs), the IL-18 system, defensins, and markers potentially associated with neuroinflammation and BBB integrity; detailed single marker case-control analyses of several of the factors, i.e. CAMs, NSE, BAFF, APRIL and IL-18 markers are reported previously in overlapping samples (Andreou et al., 2021; Engh et al., 2021; Sheikh et al., 2022; Szabo et al., 2022). The chemokines were growth-regulated oncogene alpha (GROα), stromal cell-derived factor 1 alpha (SDF1α), eotaxin, and regulated upon activation, normal T-cell expressed and secreted (RANTES) (Louboutin and Strayer, 2013; Mantyla et al., 2015; Reale et al., 2011; Stuart and Baune, 2014). CAMs were mucosal vascular addressin cell adhesion molecule-1 (MadCAM-1), neural cadherin (NCAD), junctional adhesion molecule-A (JAMA), intercellular adhesion molecule-1 (ICAM-1), vascular cell adhesion molecule-1 (VCAM-1) and P-selectin (PSEL) (Sakurai, 2017; Sladojevic et al., 2014; Wu et al., 2017). The IL-18 system was assessed by IL-18, IL-18 binding protein (IL-18BP), IL-18 receptor 1 (IL-18R1) and IL-18 receptor accessory protein (IL-18RAP) (Alboni et al., 2010; Bossu et al., 2010; Cherlin et al., 2020; Dinarello et al., 2013). The defensins were human neutrophil peptides 1–3 (HNP1-3), beta defensin 1 (BD-1) and beta defensin 2 (BD-2) (Craddock et al., 2008; Hao et al., 2001; Williams et al., 2012). Markers particularly associated with BBB integrity were S100 calcium binding protein B (S100B) (Michetti et al., 2018), furin (Hou et al., 2018; Thomas, 2002), neuron specific enolase (NSE) (Haque et al., 2018) and glial fibrillary acidic protein (GFAP) (Trepanier et al., 2016), and markers with potential association with neuroinflammation were A proliferation-inducing ligand (APRIL) and B-cell-activating factor belong to the TNF family (BAFF) (Bossen and Schneider, 2006), alpha-2-macroglobulin (A2M) (Yee et al., 2017) and serpin family A member 3 (SERPINA3) (Chiu et al., 1999; Horváth and Mirnics, 2014; Ramsey et al., 2013; Saetre et al., 2007).

### 2.5 Definition of illness course characteristics

We included the following illness course characteristics:

*Number of psychotic episodes per year (lifetime), SCZ*: Patients were categorized according to the quartiles of number of psychotic episodes per year of illness duration, and the patients in the lower quartile (below the 25th percentile, 0.18 episodes/year) and in the upper quartile (above the 75th percentile, 1.00 episodes/year) were selected for statistical analysis (Cella et al., 2014; Nopoulos et al., 1998) to compare different groups in terms of prognostic value by the least and most severe illness course in terms of rate of episodes, respectively (Emsley et al., 2013), to facilitate interpretation of results and clinical relevance. Current or previous psychotic episodes were identified by the SCID-1 assessment and use of medical records.

*Number of affective episodes per year (lifetime), BD*: Patients were categorized according to the quartiles of number of affective episodes per year of illness duration, and the patients in the lower quartile (below the 25th percentile, 0.46 episodes/year) and in the upper quartile (above the 75th percentile, 1.85 episodes/year) were selected for statistical analysis (Cella et al., 2014; Nopoulos et al., 1998) to compare different groups in terms of prognostic value by the least and most severe illness course in terms of rate of episodes, respectively (Peters et al., 2016), to facilitate interpretation of results and clinical relevance. Current and previous affective episodes included hypomanic, manic, major depressive and mixed episodes, and were identified by the SCID-1 assessment and use of medical records.

*Suicide attempt (lifetime):* Patients were categorized into no history (‘absent’) or history (‘present’) of suicide attempt based on interviews and medical records.

*Comorbid substance use disorder (lifetime)*: Three variables, each with patients dichotomized into no history (current or previous) (‘absent’) or a history (current or previous) (‘present’), of 1) any comorbid substance use disorder, 2) comorbid alcohol use disorder, and 3) comorbid cannabis use disorder, respectively, were made based on the SCID-1 assessment.

*BD with psychotic episodes (lifetime):* Patients with BD were categorized into no history (current or previous) (‘absent’) or a history (current or previous) (‘present’) of psychotic episodes based on the SCID-1 assessment.

*Age at onset*: The patient subsample was categorized according to age at onset of first illness episode (psychotic, hypomanic, manic, major depressive or mixed): ‘early onset’ <18 years, ‘adult onset’ ≥18 years <40, and ‘late onset’ ≥40 years.

### 2.6 Statistical analysis

All statistical analyses were performed using the Statistical Package for the Social Sciences (SPSS) for Windows version 27 (SPSS Inc., Chicago, IL, USA). Sample characteristics were analyzed using independent *t*-test for normally distributed variables, Kruskal-Wallis test and Mann-Whitney *U* test for non-normally distributed variables and chi-square tests for categorical variables. For correlations we used Pearson's r and Spearman's rho. Normality was assessed by use of histograms, Q-Q-plots and Kolmogorov-Smirnov statistics. The immune marker data was log-transformed followed by repeated removal of residuals more extreme than 3 x IQR or 1.5 x IQR below or above the 25th and 75th percentile, respectively, depending on the distribution of the marker (3 x IQR: BAFF, APRIL, furin, GFAP, SDF1α, eotaxin, JAMA, NCAD, ICAM-1, VCAM-1, SERPINA3, IL-18BP, IL-18R1, BD-1, BD-2; 1.5 x IQR: S100B, GROα, MadCAM-1, IL-18RAP, HNP1-3).

**Fig. 1:**
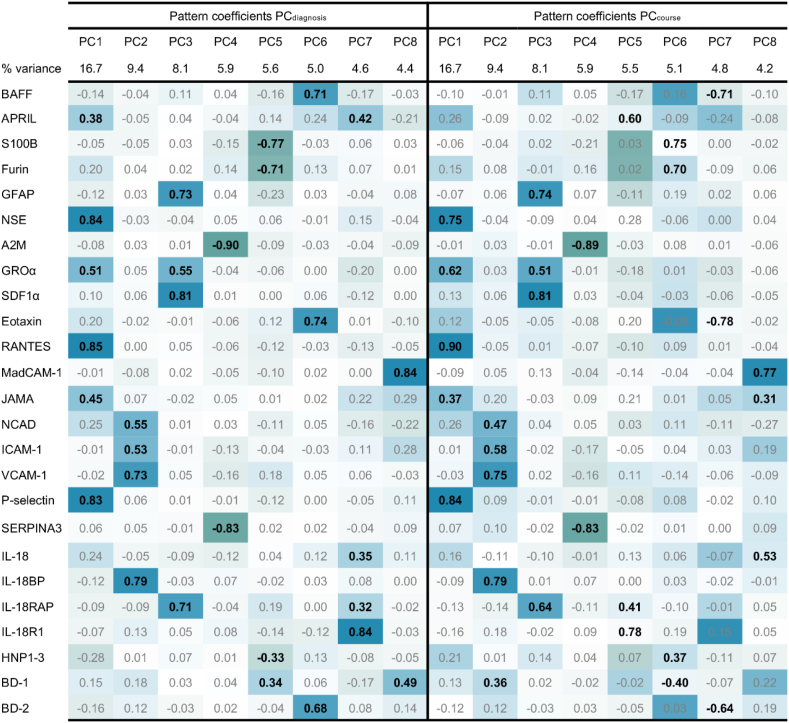
Principal component analysis^a^ in total sample (PC_diagnosis_) and patient subsample (PC_course_), pattern matrix ^a^Oblimin rotation The pattern matrix contains regression coefficients (loadings), with values ≥ 0.3 as bolded for description of PCs. Abbreviations: Principal component (PC).

*Principal Component Analyses (PCA)* of the 25 immune markers were performed to reduce the dataset into fewer variables while retaining the majority of the variance within the sample (Dickerson et al., 2015; Lindqvist et al., 2011; Nguyen et al., 2018; Raymond et al., 2019). PCA was used to identify subgroups of immune markers, principal components (PCs). PC scores (‘composite immune marker scores’) were calculated from the PCA and used in the statistical models, representing the participant's score on each component. PCA of the 25 immune markers were performed in a) the total sample of patients and HC (SCZ + BD + HC) to generate ‘PC_diagnosis_’ scores for analysis of diagnoses related immune patterns, and in b) the patient subsample (SCZ + BD) to generate ‘PC_course_’ scores for analysis of illness specific course characteristics with immune patterns. The PCA did not include other factors than the immune markers. All test assumptions (Field, 2018) were met; the Kaiser-Meyer-Olkin value was 0.749 and 0.742 for the total sample and the patient subsample, respectively, exceeding the recommended value of 0.5, indicating that a PCA is appropriate. The Bartlett's Test of Sphericity was statistically significant (p < 0.001) in both the total and the subsample, supporting the factorability of the correlation matrices. Oblimin rotation was applied to allow for correlation between components, and PCs were extracted based on an Eigenvalue cut-off of >1 (Kaiser's criterion). PCs are in the following described by immune markers with component loadings above 0.3. For complete details of immune marker component loadings of each PC, and PC raw scores, see Fig. 1 and Supplementary Table 2, respectively.

*Multivariate analyses of covariance (MANCOVAs)* were performed separately for diagnosis (SCZ, BD, HC) and each illness course characteristic (independent variables in separate MANCOVAs) to assess differences in the PC scores (dependent variables) between groups. MANCOVA was applied due to the case of several dependent variables, using an initial MANCOVA omnibus test to indicate an effect (Wilks’ lambda). To include potential confounding factors while keeping model complexity low and ensuring clarity of variable selection, analyses were performed with backward elimination of potential confounders, including age, sex, BMI and freezer storage time, and also SCZ and BD diagnosis and psychopharmacological treatment [antipsychotic use (yes/no), antidepressant use (yes/no), anticonvulsants and/or lithium (yes/no)] in patient subsample analysis. A p-value of >0.05 was used for removal of variables. See Supplementary Table 3 for details of the backward elimination. Differences in individual PC scores of dichotomous illness course characteristics were obtained from the univariate test statistics output and of age at onset and diagnosis (three categories) from the univariate tests by pairwise comparisons based on estimated marginal means. Given the sparsely explored research area, a modest correction of the significance level threshold to p < 0.025 (0.05/2) was applied for the initial MANCOVA omnibus test based on analyzing 1) diagnosis and 2) illness course characteristics, to indicate variables for further analysis while avoiding excessive risk of rejecting hypotheses that merit further investigation. In the follow-up univariate analyses of the indicated variables and each of the eight PCs, the significance level threshold was Bonferroni corrected to p < 0.00625 (0.05/8) due to analyzing eight PCs, and pairwise comparisons were controlled by standard method of substituting p-values lower than the omnibus test p-value, with the omnibus test p-value (Levin et al., 1994). All p-values are reported uncorrected.

### 2.7 Ethics

The TOP study is approved by the Regional Scientific Ethical Committee and the Norwegian Data Protection Inspectorate. The Biobank is approved by the Norwegian Directorate of Health. Participation is voluntary and written consent is a prerequisite. Information on the study and the possibility to withdraw is given both written and orally.

## 3 Results

### 3.1 Demography, symptoms, illness course characteristics, immune markers and principal components

There were more female participants in the BD group as compared to the SCZ and HC groups (both p < 0.001), and participants with SCZ were younger than those with BD and HC (both p < 0.001). Participants with SCZ were more severely ill as measured by GAF and PANSS (both p < 0.001) and had less frequent early onset (p < 0.001) and comorbid alcohol use disorder (p = 0.002) compared to the BD group, see Table 1 for details. For concentrations of the 25 immune markers across groups, see Supplementary Table 4. Immune marker differences in overlapping samples were reported in previous publications (Andreou et al., 2021; Engh et al., 2021; Sheikh et al., 2022; Szabo et al., 2022). Bivariate correlations between sample characteristics and PCs are given in Supplementary Table 6.

**Table 1:** Demographic and illness course characteristics.

	Categories	SCZ (N = 602)	BD (N = 346)	HC (N = 814)	*p*-value^c^	Pairwise comparisons
**Sample characteristics**						
Sex, males, N (%)		358 (59.5)	142 (41.0)	456 (56.0)	**<0.001**	BD < SCZ,HC
Age (years), median (IQR)		27 (22–36)	31 (23–42)	32 (26–39)	**<0.001**	SCZ < BD,HC
BMI (kg/m2), median (IQR)		25.1 (22.4–29.1)	25.1 (22.3–27.7)	24.1 (22.1–26.4)	**<0.001**	HC < SZ,BD
Freezing time^a^ (years), median (IQR)		8 (5–10)	8 (3–10)	5 (3–9)	**<0.001**	HC < SZ,BD
GAF-S, median (IQR)		40 (37–51)	58 (51–65)	–	**<0.001**	–
PANSS, median (IQR)		61 (51–71)	43 (38–51)	–	**<0.001**	–
Duration of illness (years), median (IQR)		4.0 (1–10)	10 (5–19)	–	**<0.001**	BD < SCZ
Antidepressant use, N (%)		169 (28.1)	115 (33.2)	–	0.11	–
Antipsychotic use, N (%)		508 (84.4)	192 (55.5)	–	**<0.001**	–
Anticonvulsant and lithium use, N (%)		74 (12.3)	181 (52.3)	–	**<0.001**	–
**Illness course characteristics**						
Age at onset, N (%)	<18/18–39/>40	131 (22.5)/422 (72.5)/29 (5.0)	149 (43.2)/181 (52.5)/15 (4.3)	–	**<0.001**	–
Psychotic episodes, SCZ, N^b^	upper/lower	120/117	–	–		–
Affective episodes, BD, N^b^	upper/lower	–	78/79	–		–
Suicide attempt, N (%)	present/absent	232 (45.3)/280 (54.7)	118 (39.5)/181 (60.5)	–	0.11	–
Any comorbid substance use disorder, N (%)	present/absent	140 (23.3)/462 (76.7)	80 (23.1)/266 (76.9)	–	1.0	–
Comorbid alcohol use disorder, N (%)	present/absent	23 (4.7)/462 (95.3)	32 (10.7)/266 (89.3)	–	**0.002**	–
Comorbid cannabis use disorder, N (%)	present/absent	40 (8.0)/462 (92.0)	20 (7.0)/266 (93.0)	–	0.72	–
BD with psychotic episodes, N (%)	present/absent	–	207 (61.4)/130 (38.6)	–		–

Missing data (%), sample characteristics: BMI 15.2, duration of illness 2.2; illness course characteristics: psychotic episodes of SCZ 25.6, comorbid alcohol use disorder 17.4, comorbid cannabis disorder 16.9, suicide attempt 14.5, any comorbid substance use disorder 13.1, affective episodes, BD, 9.2, BD with psychotic episodes 2.6, age at onset 2.2.

Abbreviations: Bipolar disorder (BD), Body mass index (BMI), Global Assessment of Functioning - symptoms (GAF-S), Healthy controls (HC), Interquartile range (IQR), Positive and Negative Syndrome Scale (PANSS), Schizophrenia spectrum disorders (SCZ), Severe mental disorders (SMD).

a Freezing time of plasma sample used to analyze immune markers. Minimum and maximum values (years): SCZ, 1–15; BD, 1–15; HC 1–14.

b Number of patients in the ‘upper’ and in the ‘lower’ quartile of number of psychotic (SCZ) and affective (BD) episodes per year of illness duration.

c Chi square test for categorical variables, Kruskal Wallis and Mann-Whitney *U* Test for variables represented by median (interquartile range).

The PCA reduced the 25 immune markers into different sets of eight PCs (Eigenvalue cut-off of >1) in the total sample and patient subsample, explaining 59.7% of the variances in both samples. *Total sample* PCs included the following immune markers: RANTES, NSE, PSEL, JAMA, GROα and APRIL (PC1_diagnosis_); IL-18BP, VCAM-1, NCAD and ICAM-1 (PC2_diagnosis_); SDF1α, GFAP, IL-18RAP and GROα (PC3_diagnosis_); A2M and SERPINA3 (PC4_diagnosis_); S100B, furin, HNP1-3 and BD-1 (PC5_diagnosis_); eotaxin, BAFF and BD-2 (PC6_diagnosis_); IL-18R1, APRIL, IL-18 and IL-18RAP (PC7_diagnosis_); MadCAM-1 and BD-1 (PC8_diagnosis_). *Patient subsample* PCs included the following immune markers: RANTES, PSEL, NSE, GROα and JAMA (PC1_course_); IL-18BP, VCAM-1, ICAM-1, NCAD and BD-1 (PC2_course_); SDF1α, GFAP, IL-18RAP and GROα (PC3_course_); A2M and SERPINA3 (PC4_course_); IL-18R1, APRIL and IL-18RAP (PC5_course_); S100B, furin, BD-1 and HNP1-3 (PC6_course_); eotaxin, BAFF and BD-2 (PC7_course_); MadCAM-1, IL-18 and JAMA (PC8_course_). For more details, see Fig. 1 and Supplementary Fig. 1.

### 3.2 Associations between diagnosis, age at onset and illness course characteristics and immune principal components

#### 3.2.1 Diagnosis

**Fig. 2:**
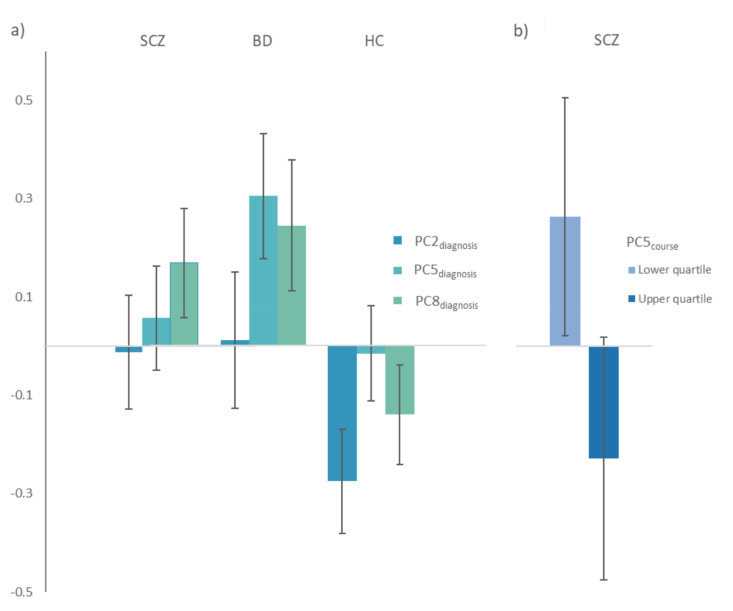
Immune principal components (PC) associated with a) diagnosis and b) number of psychotic episodes per year in SCZ a) Estimated marginal means of PC scores based on Log-10 transformed immune marker values of PC2_diagnosis_ (IL-18BP, VCAM-1, NCAD, ICAM-1), PC5_diagnosis_ (S100B, furin, HNP1-3, BD-1) and PC8_diagnosis_ (MadCAM-1, BD-1). Error bars represent confidence intervals. b) Estimated marginal means of PC5_course_ (IL-18R1, APRIL and IL-18RAP) scores based on Log-10 transformed immune marker values, of patients with SCZ in the lower and upper quartiles, respectively, of number of psychotic episodes per year. Error bars represent confidence intervals.

As shown in Table 2, there was a significant effect of *diagnosis* (SCZ, BD, HC) in the MANCOVA omnibus test of eight PC_diagnosis_ scores: F (16, 1480) = 4.692, p < 0.001, Wilks’ lambda = 0.906, partial η^2^ = 0.048. In follow-up analyses of the individual PCs, we found significant differences between diagnostic groups for PC2_diagnosis_ score: SCZ > HC (p = 0.001) and BD > HC (p = 0.001), PC5_diagnosis_ score: BD > HC (p < 0.001) and BD > SCZ (p = 0.004), and PC8_diagnosis_ score: SCZ > HC (p < 0.001) and BD > HC (p < 0.001). See Fig. 2a) and Supplementary Table 7 for details.

**Table 2:** MANCOVA omnibus tests of associations between diagnosis and eight principal components (adjustments with backward elimination procedure).

Model	Wilks' λ	F	df	Error df	*p*-value	Partial η^2^
Diagnosis^a^	0.906	4.692	16	1480	**<0.001**	0.048
Age	0.942	5.714	8	740	<0.001	0.058
Sex	0.954	4.500	8	740	<0.001	**0.046**
BMI	0.958	4.099	8	740	<0.001	0.042
Freezing time	0.684	42.820	8	740	<0.001	0.316

MANCOVA was performed for diagnosis (SCZ, BD, HC; independent variable) to assess differences in the principal component scores (eight, dependent variables).

Abbreviations: Bipolar spectrum disorder (BD), Multivariate analyses of covariance (MANCOVA), Severe mental disorders (SMD), Schizophrenia spectrum disorder (SCZ).

a Total sample (SCZ, BD, HC).

#### 3.2.2 Age at onset and illness course characteristics

There was a significant effect of *number of psychotic episodes per year, SCZ* in the MANCOVA omnibus test of the eight PC_course_ scores: F (8, 96) = 2.340, *p* = 0.024, Wilks’ lambda = 0.837, partial η^2^ = 0.163 (Table 3). The univariate test statistics showed a significant effect on PC5_course_ score: lower quartile > upper quartile (mean difference 0.493, p = 0.006). No statistically significant effects were found in MANCOVAs (omnibus test) for age at onset (p = 0.097), number of affective episodes per year, BD (p = 0.398), suicide attempt, lifetime (p = 0.831), any comorbid substance use disorder (p = 0.085), comorbid alcohol use disorder (p = 0.637), comorbid cannabis use disorder (p = 0.203), or BD with psychotic episodes (p = 0.388). See Table 3, Fig. 2b) and Supplementary Table 7 for details.

**Table 3:** MANCOVA omnibus tests of associations between illness course characteristics and eight principal components (adjustments with backward elimination procedure).

Model	Wilks' λ	F	df	Error df	*p*-value	Partial η^2^
*Age at onset*^a^	0.946	1.487	16	848	0.097	0.027
Age	0.936	3.635	8	424	<0.001	0.064
Sex	0.952	2.652	8	424	0.008	0.048
BMI	0.942	3.255	8	424	0.001	0.058
Freezing time	0.632	30.890	8	424	<0.001	0.368
*Psychotic episodes, SCZ*^a^^,^^b^	0.837	2.340	8	96	**0.024**	0.163
Freezing time	0.657	6.276	8	96	<0.001	0.343
*Affective episodes, BD*^a^^,^^b^	0.902	1.062	8	78	0.398	0.098
Sex	0.764	3.016	8	78	0.005	0.236
Freezing time	0.644	5.380	8	78	<0.001	0.356
Mood stabilizers	0.809	2.299	8	78	0.029	0.191
*Suicide attempt*^a^^,^^c^	0.989	0.534	8	401	0.831	0.011
Age	0.933	3.605	8	401	<0.001	0.067
Sex	0.945	2.909	8	401	0.004	0.055
BMI	0.929	3.822	8	401	<0.001	0.071
Freezing time	0.667	25.029	8	401	<0.001	0.333
*Any comorbid substance use disorder*^a^^,^^c^	0.970	1.752	8	448	0.085	0.030
Age	0.936	3.857	8	448	<0.001	0.064
Sex	0.943	3.394	8	448	<0.001	0.057
BMI	0.940	3.552	8	448	<0.001	0.060
Freezing time	0.617	34.733	8	448	<0.001	0.383
*Comorbid alcohol use disorder*^a^^,^^c^	0.983	0.761	8	362	0.637	0.017
Age	0.926	3.612	8	362	<0.001	0.074
Sex	0.932	3.313	8	362	0.001	0.068
BMI	0.934	3.216	8	362	0.002	0.066
Freezing time	0.619	27.835	8	362	<0.001	0.381
*Comorbid cannabis use disorder*^a^^,^^c^	0.970	1.381	8	363	0.203	0.030
Age	0.920	3.921	8	363	<0.001	0.080
Sex	0.932	3.298	8	363	0.001	0.068
BMI	0.923	3.781	8	363	<0.001	0.077
Freezing time	0.626	27.121	8	363	<0.001	0.374
*BD with psychotic episodes*^d^	0.951	1.068	8	167	0.388	0.049
Age	0.888	2.626	8	167	0.010	0.112
Sex	0.906	2.162	8	167	0.033	0.094
Freezing time	0.638	11.852	8	167	<0.001	0.362

MANCOVA was performed for age at onset and each illness course characteristic (independent variable) separately to assess differences in the principal component scores (eight, dependent variables).

Abbreviations: Bipolar spectrum disorder (BD), Multivariate analyses of covariance (MANCOVA), Severe mental disorders (SMD), Schizophrenia spectrum disorder (SCZ).

a Patient subsample (SCZ, BD).

b Number of psychotic (SCZ) and affective (BD) episodes per year of illness duration.

c Lifetime.

d BD subsample.

## 4 Discussion

PCA was used in a large SMD sample to investigate patterns of immune markers associated with diagnosis and illness course characteristics. After reduction of 25 markers into eight components, three components based on CAMs, BBB markers and defensins were associated with SCZ or BD. For course characteristics in SCZ, number of psychotic episodes per year was associated with a component of APRIL and soluble IL-18 receptor markers. Thus, our study suggests that CAMs assisted BBB dysfunction, pro-inflammatory signaling and dysregulated neuroprotective processes may play a role in the progression of SMDs.

Supporting a role of inflammation in SMDs (Goldsmith et al., 2016; Kroken et al., 2018), several immune patterns (PC_diagnosis_) were associated with a diagnosis of SCZ or BD. Using composite scores, the susceptibility of unforeseen variability of single markers is limited. A similar approach was applied by Nguyen et al. (Nguyen et al., 2018) showing an enhanced index of ICAM-1 and VCAM-1 in SCZ compared to HC. Our results in a several times larger sample extend these findings showing an association of SCZ with components strongly influenced by CAMs (PC2_diagnosis_ and PC8_diagnosis_) with a similar pattern in BD.

Specifically, ICAM-1, VCAM-1 and NCAD loaded positively on PC2_diagnosis_; however, together with IL-18BP, ICAM-1 accounted for the main difference in increased concentrations in the patient groups. Although conflicting evidence (Futtrup et al., 2020; Kronig et al., 2005; Muller, 2019; Schwarz et al., 2000; Stefanovic et al., 2016), elevated levels of ICAM-1 are previously indicated in both SCZ (Beumer et al., 2012; Cai et al., 2020; Nguyen et al., 2018; Sheikh et al., 2022; Stefanovic et al., 2016) and BD (Pantovic-Stefanovic et al., 2018; Reininghaus et al., 2016; Schaefer et al., 2016; Turan et al., 2014). ICAM-1 has also been associated with increased symptom severity (Muller, 2019; Stefanovic et al., 2016; Turan et al., 2014) and better treatment response (Stefanovic et al., 2016) in SCZ, although not associated with course characteristics in the current study. There is conflicting evidence of VCAM-1 (Futtrup et al., 2020; Nguyen et al., 2018; Stefanovic et al., 2016); however, both ICAM-1 and VCAM-1 may be related to manic episodes (Turan et al., 2014). In a study from our centre of adolescent participants with early-onset psychosis (Wedervang-Resell et al., 2020), reduced levels of PSEL and VCAM-1 and no significant alterations of ICAM-1, MadCAM-1, JAMA or NCAD, were found. While both studies suggest involvement of CAMs in SMD pathophysiology, differences indicate the need of examining temporal patterns as well as potential variations between subgroups. Increased plasma levels of IL-18 and IL-18BP was recently demonstrated in our group, as also shown by others (Palladino et al., 2012), together with higher expression of the inflammasome-related genes NLRP3 and NLRC4 in blood leukocytes in SMDs (Guo et al., 2015; Strowig et al., 2012; Szabo et al., 2022), supporting systemic inflammasome activation in these patients. IL-18BP, regulated by a negative feedback from IL-18 (Dinarello et al., 2013; Palladino et al., 2012), may therefore reflect long standing low-grade inflammasome activation in SMDs. Our finding that IL-18BP loaded positively on PC2_diagnosis_ together with CAMs expressed on vascular cells including the BBB (Marchetti and Engelhardt, 2020), may link NLRP3 and NLRC4 inflammasome activation with adhesion and transmigration of leukocytes to underlying tissues, potentially a mechanism promoting neuroinflammation in SMDs (Herman and Pasinetti, 2018).

Further support linking IL-18 signaling to SCZ was the finding that PC5_course_ with positive loadings of IL-18R1, IL-18RAP and APRIL was negatively associated with severity of illness course as measured by rate of psychotic episodes, in these patients. While the mechanism of IL-18 and IL-18BP are well established, the regulation and function of soluble IL-18R1 and IL-18RAP are less established. Membrane bound IL-18R1 and IL-18R2 promotes IL-18 signaling and is further amplified by IL-18RAP (Yang, 2020). Conversely, their soluble forms act as decoy receptors and inhibit IL-18 signaling (Reznikov et al., 2002). Thus, we speculate that within patients with SCZ, decreased levels of soluble IL-18R1 and IL-18RAP could promote IL-18 signaling at the cellular level (Alboni et al., 2010; Cherlin et al., 2020; Tsutsumi et al., 2014) and influence illness course severity. The positive loading of APRIL, a potential neuroprotective cytokine by mediating production of the anti-inflammatory IL-10 (Baert et al., 2019), in PC5_course_ in relation to less illness severity seem in line with our recently reported association of lower APRIL with increased psychotic symptoms and generally decreased levels in SMD (Engh et al., 2021). Taken together, increases in these markers with anti-inflammatory function seem to be associated with a less severe illness course.

Interestingly, PC5_diagnosis_ score was increased in BD relative to SCZ and HC with negative loadings of BBB-related markers dominating the component. While CAMs regulate migration, the major PC5_diagnosis_ marker, S100B, is an astrocyte-expressed protein indicating BBB disruption (Futtrup et al., 2020) due to e.g. brain trauma and cerebrovascular diseases (Chong et al., 2016). Several studies report elevated S100B in BD (Bartoli et al., 2020; da Rosa et al., 2016) and SCZ (Aleksovska et al., 2014; Hong et al., 2016; Rothermundt et al., 2009). However, there is a positive association with symptom severity (Futtrup et al., 2020; Pollak et al., 2018; van de Kerkhof et al., 2014) and reduced levels in BD after treatment of manic phase, suggesting a transient disruption of BBB that may be clinically dependent (Tsai and Huang, 2017). Importantly, the current patients had relatively low symptom load, enabling putative assessment of the basal underlying BBB integrity of BD. We found that furin and the alpha-defensins HNP1-3 all loaded negatively together with S100B, and BD-1 had a small positive effect. While furin is a convertase activating a range of protein precursors implicated amongst others in BBB disruption (Baumann et al., 2019), alpha-defensins have broad functions including inflammation regulatory effects such as chemotaxis (Fruitwala et al., 2019), and the current HNP1-3 markers have been linked to immunological components of a related illness, Alzheimer's disease (DeMichele-Sweet et al., 2021; Szekeres et al., 2016). Nevertheless, a BD specific BBB integrity, after comparison with SCZ and HC, is suggested by the component, a finding that warrant further investigation given the sparsity of studies of BBB markers in BD (Futtrup et al., 2020).

The defensin BD-1 constituted a positive load together with MadCAM-1 on PC8_diagnosis_. Beta-defensins have a wide repertoire of mechanisms including pro-inflammatory and anti-inflammatory functions, and may be reduced in patients with inflammatory disorders (Shelley et al., 2020) and associated with neuroinflammation in Alzheimer's disease (Williams et al., 2013). Generally, the underlying mechanisms indicated across BD and SCZ by the component loadings of PC2_diagnosis_ and PC8_diagnosis_, are pro-inflammatory processes including neuroinflammation mediated by CAMs. While CAMs are involved in various functions (Harjunpaa et al., 2019), they regulate migration of inflammatory cells across the BBB (Kong et al., 2018; Muller, 2019) and thus the current associations are indicative of BBB dysregulation with central inflammatory infiltration.

The use of composite immune variables rather than single immune markers has been proposed as a way to advance the field, both in terms of providing a coherent understanding of the interplay between immune pathways and by improving the quality of immune variable signals (Miller and Goldsmith, 2017). As suggested by the current study, analyzing immune patterns might gain additional information beyond single marker analyses, exemplified here by 1) the indicated mediating mechanism of neuroinflammation of NLRP3 and NLRC4 inflammasome linked CAMs assistance of leucocyte transmigration, 2) displaying links between BBB integrity markers in BD, and 3) suggesting interplay of different anti-inflammatory pathways in illness course severity*.* Thus, the current study adds novel information regarding interrelationships of immune pathways in SMD to the corresponding detailed studies of single immune markers of our centre demonstrating aberrations of some of the current immune markers (Andreou et al., 2021; Engh et al., 2021; Sheikh et al., 2022; Szabo et al., 2022), as well as generally to immune research in SMD (Kroken et al., 2018). In the long term, more knowledge about these and other immune interplays might enhance our ability to test hypotheses in experimental designs, broaden the targets for intervention, and hopefully repurpose and develop agents of clinical significance.

There are limitations to consider. The cross-sectional design limits interpretation about causality. For several participants the freezing time was rather long and samples from HCs were generally gathered more recently than samples from patients. Although plasma was stored at standard −80 °C and freezing time was included in the statistical models, an influence on the results related to degradation of markers with time, cannot be excluded. The immune markers are measured peripherally, complicating inferences about central mechanisms. However, the study involves centrally expressed factors and a uniquely large sample relative to other immune – SMD samples. Furthermore, given well-evidenced immune alternations in SMDs, we performed two PCAs to allow comparisons across SMDs and HC as well as analyses of illness course characteristics with immune patterns within SMDs. This prevents analyses of case-control differences of PCs associated with illness course characteristics. While the large sample size allows detection of minor effects, the effect of immune factors within the complex interplay of biological and environmental factors is expected to be small. By using immune principal components, novel findings of immune pathway interactions indicating biological mechanisms might be possible, and the PCA provide a well-established and simple method for this purpose. Also, severe illness courses due to longstanding, but few episodes, are not detected by the current course characteristics number of psychotic and affective episodes. A range of confounder variables were included in the analyses, including BMI and psychotropic medication, however, several factors might impact immune marker levels and cause residual confounding, including clinical features, smoking and cardiometabolic conditions and treatments. Moreover, immune molecules are synthesized in various tissues such as in muscles, liver, fat, the vasculature, the brain, lymphoid tissue and white blood cells; generally, this might underlie difficulties of replicating immune results. Lastly, we applied a moderate correction for multiple comparisons of the initial MANCOVA omnibus test due to testing two clinical categories, illness course characteristics and diagnosis, while adjusting for eight PCs in the follow-up univariate analyses. Importantly, SCZ and BD have previously established immune aberrations related to the component markers, and a too strict correction would risk discarding interesting hypotheses that merit further investigation.

In the current study we established immune components based on PCA of a set of immune markers to uncover immune patterns reflecting underlying mechanisms of SMDs. CAMs-mediated dysregulation of BBB integrity was suggested across BD and SCZ, and involvement of pro-inflammatory processes associated with dysregulated neuroprotective mechanisms in illness course severity in SCZ. These findings emphasize the importance of investigating multiple immune markers simultaneously to elucidate the underlying immune dynamics in SMDs.

## Declaration of competing interest

The authors declare the following financial interests/personal relationships which may be considered as potential competing interests:

Ole A. Andreassen reports a relationship with HealthLytix that includes: consulting or advisory. Ole A. Andreassen reports a relationship with Lundbeck that includes: speaking and lecture fees. Ole A. Andreassen reports a relationship with Sunovion that includes: speaking and lecture fees.
