# Supplementary material for: Composite immune marker scores associated with severe mental disorders and illness course
Source: Brain Behav Immun Health. 2022 Jul 2;24:100483. doi: 10.1016/j.bbih.2022.100483 (PMC9287150; doi:10.1016/j.bbih.2022.100483)
Supplement: Multimedia component 1 [file mmc1.docx]

**Ormerod MBEG et al., Composite immune marker scores associated with severe mental disorders and illness course**

Supplementary material

| Supplementary Table 1. Somatic medication use by patients | | |
| --- | --- | --- |
| Somatic medications | SCZ (N=602) | BD (N=346) |
| Anti-inflammatory/immunomodulatory, N (%) | 17 (2.8) | 8 (2.3) |
| Antidiabetics, N (%) | 10 (1.66) | 4 (1.2) |
| Cardiovascular/lipid modifying, N (%) | 19 (3.16) | 15 (4.3) |
| Gastrointestinal drugs, N %) | 21 (3.5) | 8 (2.3) |
| Other, N (%) | 86 (14.3) | 74 (21.4) |

| Supplementary Table 2. Principal component (PC) raw scores | | | | | |
| --- | --- | --- | --- | --- | --- |
|  | SCZ (N=602) | BD (N=346) | HC (N=814) | p-value^a^ |  |
| Total sample | |  |  |  |  |
| PC1_diagnosis_ score | -0.226 (1.01) | -0.158 (0.98) | -0.027 (0.93) | 0.02 |  |
| PC2 _diagnosis_ score | -0.004 (1.00) | -0.011 (1.01) | -0.198 (0.89) | 0.01 |  |
| PC3 _diagnosis_ score | -0.346 (0.80) | -0.274 (0.85) | -0.205 (0.89) | 0.10 |  |
| PC4 _diagnosis_ score | -0.020 (1.00) | -0.141 (0.95) | -0.022 (0.97) | 0.32 |  |
| PC5 _diagnosis_ score | 0.141 (0.91) | 0.346 (0.92) | 0.186 (0.98) | <0.001 |  |
| PC6 _diagnosis_ score | -0.205 (0.90) | -0.208 (0.90) | -0.176 (0.93) | 0.89 |  |
| PC7 _diagnosis_ score | 0.141 (0.91) | 0.137 (0.87) | 0.035 (0.88) | 0.22 |  |
| PC8 _diagnosis_ score | 0.135 (0.94) | 0.244 (0.93) | -0.070 (0.97) | <0.001 |  |
| Patient subsample | | |  |  |  |
| PC1_course_ score | -0.143 (1.00) | -0.083 (0.97) | - | 0.51 |  |
| PC2 _course_ score | -0.120 (0.92) | -0.093 (0.95) | - | 0.76 |  |
| PC3 _course_ score | -0.290 (0.82) | -0.230 (0.88) | - | 0.45 |  |
| PC4 _course_ score | -0.022 (0.98) | -0.157 (0.93) | - | 0.14 |  |
| PC5 _course_ score | -0.011 (0.92) | 0.078 (0.86) | - | 0.29 |  |
| PC6 _course_ score | -0.011 (0.94) | -0.365 (0.93) | - | <0.001 |  |
| PC7 _course_ score | 0.213 (0.91) | 0.213 (0.89) | - | 0.99 |  |
| PC8 _course_ score | 0.049 (0.98) | 0.060 (0.92) | - | 0.90 |  |
| Mean (SD) PC scores based on Log10-transformed immune marker values ^a^One-way ANOVA or Independent T-test | | | | | |

| Supplementary Table 3. Backward elimination^a^ in MANCOVA analyses | | | | | | | | |
| --- | --- | --- | --- | --- | --- | --- | --- | --- |
|  | **Sex** | **Age** | **Diagnosis** | **BMI** | **FT** | **AD** | **AP** | **MS** |
| Diagnosis^b^ |  |  |  |  |  | 1 | 2 |  |
| Age at onset^c^ |  |  | 3 |  |  | 1 | 2 |  |
| Psychotic episodes, SCZ^c,d^ | 5 | 3 |  | 4 |  | 2 | 1 |  |
| Affective episodes, BD^c,d^ |  | 3 |  | 1 |  | 2 | 4 |  |
| Suicide attempt^c,e^ |  |  | 1 |  |  | 2 | 3 |  |
| Any comorbid substance use disorder^c,e^ |  |  | 3 |  |  | 1 | 2 |  |
| Comorbid alcohol use disorder^c,e^ |  |  | 3 |  |  | 1 | 2 |  |
| Comorbid cannabis use disorder^c,e^ |  |  | 3 |  |  | 1 | 2 |  |
| BD with psychotic episodes^f^ |  |  |  | 2 |  | 1 | 3 | 4 |
| ^a^Backward eliminations by excluding variables with p-value > 0.05 from MANCOVA: 1 = 1^st^ adjustment, 2 = 2^nd^ adjustment, 3 = 3^rd^ adjustment, 4 = 4^th^ adjustment, 5 = 5^th^ adjustment  ^b^Total sample (SCZ, BD, HC)  ^c^Patient subsample (SCZ, BD)  ^d^Number of psychotic (SCZ) and affective (BD) episodes per year of illness duration  ^e^Lifetime  ^f^BD subsample  Abbreviations: Anticonvulsant agent or Lithium use (‘mood stabilizers’, MS), Antidepressant agent use (AD), Antipsychotic agent use (AP), Bipolar spectrum disorder (BD), Body mass index (BMI), Freezer storage time (FT), Multivariate analysis of covariance (MANCOVA), Severe mental disorders (SMD), Schizophrenia spectrum disorder (SCZ) | | | | | | | | |

| Supplementary Table 4. Immune markers associated with groups^*^ | | | | | | |
| --- | --- | --- | --- | --- | --- | --- |
| **Immune markers^a^** | **SCZ (N = 602)** | **BD (N = 346)** | **HC (N = 814)** | ***p*-value^b^** | **Pairwise comparisons^b^** |  |
| *Neuroinflammation* |  |  |  |  |  |  |
| BAFF (pg/mL) | 234 (178, 311) | 215 (177, 292) | 220 (173, 293) | 0.11 | - |  |
| APRIL (pg/mL) | 249 (153, 335) | 254 (166, 386) | 317 (217, 455) | **1.8x10^-11^** | BD, SCZ<HC |  |
| A2M (µg/mL) | 13.8 (9.24, 20.5) | 15.3 (10.4, 21.1) | 13.6 (9.53, 18.2) | 0.39 | - |  |
| SA3 (µg/mL) | 1.21 (0.83, 1.70) | 1.35 (0.92, 1.82) | 1.25 (0.87, 1.65) | **0.03** | HC, SCZ<BD |  |
| Sum, z-score | -0.51 (-1.54, 0.84) | -0.26 (-1.43, 1.02) | -0.58 (-1.64, 0.81) | 0.21 | - |  |
| *BBB integrity* |  |  |  |  |  |  |
| S100B (ng/mL) | 101 (91, 115) | 98 (89, 110) | 103 (92, 115) | **0.001** | BD<SCZ, HC |  |
| Furin (ng/mL) | 0.38 (0.28, 0.51) | 0.34 (0.25, 0.46) | 0.34 (0.27, 0.48) | **0.002** | BD<SCZ, HC |  |
| GFAP (pg/mL) | 134 (99.1, 187) | 137 (101, 198) | 134 (95, 193) | 0.73 | - |  |
| NSE (ng/mL) | 2.41 (1.09, 4.82) | 2.85 (1.24, 6.06) | 2.92 (1.37, 6.29) | **2.7x10^-5^** | SCZ, BD<HC |  |
| Sum, z-score | -0.68 (-1.47, 0.51) | -0.78 (-1.53, 0.30) | -0.55 (-1.37, 0.77) | **0.04** | BD<HC |  |
| *Chemokines* |  |  |  |  |  |  |
| GROα (pg/mL) | 18.9 (12.9, 27.2) | 18.5 (12.9, 26.1) | 19.7 (14.4, 26.9) | **0.003** | BD, SCZ<HC |  |
| SDF1α (pg/mL) | 1357 (1090, 1709) | 1431 (1110, 1844) | 1463 (1149, 1881) | **0.003** | SCZ<HC |  |
| Eotaxin (pg/mL) | 131 (91.5, 177) | 126 (96.6, 162) | 135 (105, 183) | **3.5x10^-4^** | SCZ, BD<HC |  |
| RANTES (ng/mL) | 75.9 (50.0, 117) | 73.8 (49.4, 114.7) | 85.0 (54.9, 119.4) | **0.0002** | SCZ, BD<HC |  |
| Sum, z-score | -0.76 (-1.62, 0.40) | -0.79 (-1.63, 0.54) | -0.36 (-1.37, 1.00) | **3.8x10^-5^** | SCZ, BD<HC |  |
| *Cell adhesion molecules* |  |  |  |  |  |  |
| MadCAM-1 (ng/mL) | 7.71 (6.04, 10.13) | 7.57 (5.95, 9.50) | 7.21 (5.55, 9.00) | **2.0x10^-6^** | HC<SCZ, BD |  |
| JAMA (ng/mL) | 1.25 (0.97, 1.71) | 1.34 (0.99, 1.75) | 1.21 (0.87, 1.63) | **0.002** | HC<SCZ, BD |  |
| NCAD (ng/mL) | 6.40 (5.62, 7.99) | 6.72 (5.70, 7.83) | 6.38 (5.54, 7.60) | 0.31 | - |  |
| ICAM-1 (ng/mL) | 271 (221, 337) | 275 (221, 335) | 254 (206, 304) | **2.1x10^-9^** | HC<SCZ, BD |  |
| VCAM-1 (ng/mL) | 484 (413, 569) | 494 (410, 586) | 496 (428, 566) | 0.39 | - |  |
| PSEL (ng/mL) | 41.1 (29.5, 59.5) | 40.3 (30.4, 58.2) | 43.6 (30.3, 60.3) | 0.31 | - |  |
| Sum, z-score | -0.21 (-1.87, 1.89) | -0.46 (-1.90, 1.73) | -0.88 (-2.19, 0.87) | **0.0001** | HC<SCZ, BD |  |
| *IL-18 system* |  |  |  |  |  |  |
| IL-18 (pg/mL) | 976 (446, 1868) | 774 (359, 1861) | 791 (317, 1596) | **4.9x10^-5^** | HC<SCZ |  |
| IL-18BP (ng/mL) | 6.10 (4.93, 7.65) | 5.93 (4.90, 7.18) | 5.48 (4.47, 6.63) | **1.1x10^-16^** | HC<SCZ, BD; BD<SCZ |  |
| IL-18RAP (pg/mL) | 44.5 (40.4, 52.7) | 45.9 (41.8, 54.6) | 46.3 (41.3, 54.6) | **0.036** | SCZ<HC |  |
| IL-18R1 (ng/mL) | 0.85 (0.66, 1.12) | 0.86 (0.67, 1.08) | 0.82 (0.66, 1.01) | **2x10^-6^** | HC<SCZ, BD; BD<SCZ |  |
| Sum, z-score | -0.08 (-1.05, 1.41) | -0.30 (-1.27, 0.73) | -0.72 (-1.52, 0.29) | **2.2x10^-12^** | HC<SCZ, BD; BD<SCZ |  |
| *Defensins* |  |  |  |  |  |  |
| HNP1-3 (ng/mL) | 7.25 (5.97, 9.43) | 6.75 (5.93, 8.23) | 7.09 (6.10, 9.52) | **0.047** | BD<HC |  |
| BD-1 (ng/mL) | 11.8 (9.11, 14.11) | 12.9 (10.4, 16.6) | 12.4 (10.2, 14.9) | **1.0x10^-4^** | SCZ<BD, HC; BD<HC |  |
| BD-2 (ng/mL) | 257 (167, 419) | 253 (163, 382) | 215 (132, 373) | **1.2x10^-7^** | HC<SCZ, BD |  |
| Sum, z-score | -0.46 (-1.10, 0.31) | -0.17 (-0.92, 0.84) | -0.48 (-1.05, 0.41) | **0.009** | SCZ, HC<BD |  |
| ^a^Median (interquartile range)  ^b^Kruskal-Wallis test and Mann-Whitney *U-*test  Sum, z-score: Sum of standard scores (z-scores) of markers  Percentage below detection limit (%): GROα 9.9, Eotaxin 0.06, Furin 0.6  Missing data (%): BAFF 0, APRIL 0, S100B 0.3, Furin 0.3, GFAP 0.3, ENO2 0.06, A2M 0.02, GROα 0, SDF1α 0, Eotaxin 0.06, RANTES 0, MadCAM-1 0.06, JAMA 0, NCAD 0, ICAM-1 0, VCAM-1 0.2, PSEL 0.2, SA3 0.2, IL-18 2.3, IL-18BP 0.06, IL-18RAP 0.3, IL-18R1 1.9, HNP1-3 0.06, BD-1 0.2, BD-2 0  Abbreviations: Bipolar disorder (BD), Healthy controls (HC), Schizophrenia spectrum disorder (SCZ)  ^*^Reported elsewhere (Andreou et al., 2021; Engh et al., 2021; Sheikh et al., 2022; Szabo et al., 2022) | | | | | | |

| Supplementary Table 5. Immunoassay details and characteristics | | | | | | | | | |
| --- | --- | --- | --- | --- | --- | --- | --- | --- | --- |
|  | Company | Item# | Intra CV | Inter CV | Sensitivity* | Diurnal % | p= | Postprandial % | p= |
| BAFF | Peprotech | Ag: 310-13  CAb: M64  Dab: P163GBT | 6.1 % | 9.1 % | 32 pg/mL | 89 % | 0.19 | 96 % | 0.72 |
| APRIL | Peprotech | Ag: 310-10C  CAb: P192  Dab: P192BT | 3.5 % | 12.4 % | 14 pg/mL | 79 % | 0.22 | 114 % | 0.38 |
| S100B | RnD systems | DY1820-05 | 3.4 % | 7.1 % | 27 pg/mL | 102 % | 0.44 | 95 % | 0.21 |
|  |  |  |  |  |  |  |  |  |  |
| Furin | RnD systems | DY1503 | 8.8 % | 6.4 % | 49 pg/mL | 102 % | 0.82 | 87 % | 0.30 |
| GFAP | RnD systems | DY2594-05 | 7.1 % | 9.2 % | 17 pg/mL | 108 % | 0.59 | 85 % | 0.24 |
| NSE | RnD systems | DY5169-05 | 4.9 % | 10.3 % | 62 pg/mL | 78 % | 0.43 | 118 % | 0.38 |
| A2M | RnD systems | DY1938 | 8.3 % | 8.5 % | 0.79 ng/mL | 107 % | 0.54 | 80 % | 0.21 |
| GROα | Peprotech | 900-K38 | 5.5 % | 9.1 % | 10 pg/mL | 89 % | 0.44 | 113 % | 0.17 |
| SDF1α | Peprotech | 900-K92 | 3.9 % | 11.2 % | 60 pg/mL | 98 % | 0.79 | 88 % | 0.10 |
| Eotaxin | Peprotech | 900-K69 | 4.9 % | 5.5 % | 12 pg/mL | 94 % | 0.30 | 96 % | 0.58 |
| RANTES | Peprotech | 900-K33 | 5.2 % | 8.9 % | 20 pg/mL | 91 % | 0.35 | 128 % | 0.079 |
| MadCAM-1 | RnD systems | DY6056-05 | 3.8 % | 9.1 % | 20 pg/mL | 99 % | 0.73 | 99 % | 0.95 |
| JAMA | Sino biological | SEKA10198 | 5.5 % | 10.3 % | 42 pg/mL | 100 % | 0.99 | 107 % | 0.66 |
| NCAD | RnD systems | [DY1388-05](https://www.rndsystems.com/products/human-n-cadherin-duoset-elisa_dy1388-05) | 2.8 % | 8.1 % | 12 pg/mL | 93 % | 0.32 | 97 % | 0.66 |
| ICAM-1 | Sino biological | SEKA10346 | 4.9 % | 8.5 % | 7 pg/mL | 95 % | 0.46 | 86 % | 0.34 |
| VCAM-1 | RnD systems | DY809 | 3.9 % | 7.6 % | 19 pg/mL | 95 % | 0.16 | 89 % | 0.11 |
| PSEL | RnD systems | [DY137](https://www.rndsystems.com/products/human-p-selectin-cd62p-duoset-elisa_dy137) | 3.5 % | 8.3 % | 15 pg/mL | 87 % | 0.24 | 109 % | 0.36 |
| SA3 | Sino biological | SEK10307 | 5.9 % | 17.9 % | 50 pg/mL | 106 % | 0.65 | 95 % | 0.76 |
| IL-18 | RnD systems | DY318-05 | 2.6 % | 8.4 % | 22 pg/mL | 97 % | 0.33 | 101 % | 0.78 |
| IL-18BP | RnD systems | [DY119](https://www.rndsystems.com/products/human-il-18-bpa-duoset-elisa_dy119) | 5.1 % | 8.4 % | 25 pg/mL | 93 % | 0.17 | 96 % | 0.37 |
| IL-18RAP | Sino biological | SEK10176 | 8.3 % | 10.6 % | 5 pg/mL | 98 % | 0.39 | 98 % | 0.31 |
| IL-18R1 | Sino biological | SEK11102 | 4.7 % | 9.6 % | 25 pg/mL | N/A |  |  |  |
| HNP1-3 | Hycult | Ag: HC4014  CAb: HM2058  Dab: HM2058BT | 3.9 % | 9.2 % | 272 pg/mL | 89 % | 0.35 | 118 % | 0.28 |
| BD-1 | Peprotech | Ag: 300-51A  CAb: P253  Dab: P253BT | 3.6 % | 8.3 % | 15 pg/mL | 97 % | 0.56 | 100 % | 0.99 |
| BD-2 | Peprotech | Ag: 300-49  CAb: P161G  Dab: P161GBT | 7.1 % | 8.1 % | 32 pg/mL | 109 % | 0.57 | 102 % | 0.66 |
| *Defined as readout of 3xSD of low level sample; Ag, antigen; CAb, capture antibody; Dab, detection antibody. Diurnal variation defined as % paired difference between non-fasting sample at 0800 and 1200, n=6. Postprandial variation defined as % paired difference between non-fasting sample at 0800 and fasting sample at 0800 next day, n=6 | | | | | | | | | |

| Supplementary Table 6. Bivariate correlation analyses between sample characteristics and principal components (PC) | | | | | | | | |
| --- | --- | --- | --- | --- | --- | --- | --- | --- |
|  | PC1 | PC2 | PC3 | PC4 | PC5 | PC6 | PC7 | PC8 |
|  | Total sample | | | | | | | |
| Sex | **-0.07 (0.05)** | **-0.11 (<0.001)** | **0.07 (0.05)** | **-0.13 (<0.001)** | **0.07 (0.04)** | -0.02 (0.60) | **-0.10 (0.01)** | -0.03 (0.44) |
| Age^a^ | 0.02 (0.66) | 0.03 (0.43) | -0.06 (0.06) | **0.15 (<0.001)** | **0.11 (<0.001)** | 0.06 (0.07) | -0.03 (0.34) | **0.16 (<0.001)** |
| BMI^a^ | **0.08 (0.03)** | 0.07 (0.06) | -0.08 (0.82) | **0.15 (<0.001)** | 0.01 (0.87) | 0.03 (0.46) | **0.12 (<0.001)** | **0.20 (<0.001)** |
| Freezing time^a^ | **-0.13 (<0.001)** | -0.05 (0.15) | **0.14 (<0.001)** | 0.02 (0.58) | **-0.43 (<0.001)** | **-0.16 (<0.001)** | **-0.37 (<0.001)** | -0.06 (0.08) |
|  | Patient subsample | | | | | | | |
| Sex | **-0.12 (0.01)** | -0.06 (0.21) | **0.10 (0.03)** | **-0.15 (<0.001)** | -0.01 (0.76) | **-0.09 (0.05)** | **0.03 (0.48)** | -0.08 (0.08) |
| Age^a^ | 0.003 (0.95) | 0.08 (0.07) | -0.02 (0.72) | **0.17 (0.001)** | -0.02 (0.70) | **-0.11 (0.02)** | -0.01 (0.76) | **0.15 (0.001)** |
| BMI^a^ | **0.14 (0.004)** | **0.10 (0.04)** | 0.02 (0.67) | **0.19 (<0.001)** | 0.04 (0.36) | 0.02 (0.67) | 0.01 (0.85) | **0.23 (<0.001)** |
| Freezing time^a^ | -0.01 (0.77) | -0.01 (0.75) | **0.19 (<0.001)** | -0.01 (0.84) | **-0.42 (<0.001)** | **0.35 (<0.001)** | **0.12 (0.01)** | **-0.21 (<0.001)** |
| GAF-S | 0.003 (0.94) | -0.14 (0.75) | 0.04 (0.41) | -0.05 (0.24) | 0.08 (0.10) | **-0.24 (<0.001)** | 0.01 (0.79) | 0.02 (0.64) |
| PANSS total^a^ | -0.05 (0.29) | 0.06 (0.21) | **-0.08 (0.09)** | 0.06 (0.18) | **-0.11 (0.01)** | **0.18 (<0.001)** | 0.003 (0.99) | -0.01 (0.89) |
| PANSS positive^a^ | -0.07 (0.11) | -0.01 (0.91) | -0.06 (0.18) | **0.10 (0.03)** | -0.08 (0.07) | **0.18 (<0.001)** | 0.05 (0.32) | 0.002 (0.97) |
| PANSS negative^a^ | -0.04 (0.40) | 0.04 (0.34) | -0.06 (0.21) | 0.08 (0.09) | **-0.10 (0.02)** | **0.19 (<0.001)** | -0.02 (0.70) | 0.0001 (0.99) |
| PANSS general^a^ | -0.05 (0.32) | 0.06 (0.17) | -0.08 (0.07) | -0.001 (0.98) | **-0.10 (0.03)** | **0.12 (0.01)** | -0.02 (0.70) | -0.02 (0.70) |
| Duration of illness^a^ | 0.01 (0.82) | 0.04 (0.41) | -0.06 (0.23) | **0.08 (0.08)** | -0.09 (0.07) | **-0.18 (<0.001)** | -0.02 (0.64) | -0.02 (0.64) |
| Antidepressant use | 0.01 (0.78) | -0.01 (0.91) | 0.01 (0.76) | -0.04 (0.34) | 0.01 (0.79) | -0.02 (0.61) | -0.06 (0.16) | -0.02 (0.63) |
| DDD Antidepressant use^a^ | 0.4 (0.64) | 0.07 (0.44) | 0.07 (0.44) | 0.02 (0.79) | 0.03 (0.70) | -0.09 (0.32) | -0.06 (0.45) | 0.08 (0.37) |
| Antipsychotic use^a^ | -0.07 (0.13) | 0.02 (0.61) | 0.01 (0.93) | -0.04 (0.43) | -0.02 (0.70) | **0.11 (0.02)** | -0.05 (0.26) | -0.02 (0.68) |
| DDD Antipsychotic use^a^ | 0.04 (0.52) | -0.04 (0.43) | 0.05 (0.35) | 0.01 (0.93) | 0.02 (0.67) | 0.10 (0.08) | -0.03 (0.60) | 0.05 (0.37) |
| Anticonvulsant and lithium use | 0.07 (0.13) | **0.12 (0.01)** | -0.06 (0.18) | 0.0003 (0.99) | 0.002 (0.96) | **-0.13 (0.003)** | 0.06 (0.22) | 0.02 (0.66) |
| DDD Anticonvulsant and Lithium use^a^ | **0.21 (0.02)** | 0.07 (0.43) | 0.17 (0.06) | 0.07 (0.45) | 0.09 (0.29) | 0.10 (0.25) | -0.10 (0.24) | 0.08 (0.39) |
| Correlations are given with Pearson's r or ^a^Spearman's rho and the *p*-value in paranthesis | | | | | | | | |

| Supplementary Table 7. Associations between diagnosis and illness course characteristics with the individual principal components (eight, dependent variable) in MANCOVA analysis | | | | | | |
| --- | --- | --- | --- | --- | --- | --- |
| **Characteristics** | **F** | **df** | **Error df** | ***p*-value** | **Partial η^2^** | **Pairwise comparisons** |
| Diagnosis^a^ (IV) | | | | | | |
| PC1_diagnosis_ | 1.860 | 2 | 747 | 0.16 | 0.005 |  |
| PC2_diagnosis_ | 7.616 | 2 | 747 | **0.001** | 0.020 | HC<BD, SCZ |
| PC3 _diagnosis_ | 3.107 | 2 | 747 | 0.045 | 0.008 |  |
| PC4 _diagnosis_ | 0.858 | 2 | 747 | 0.42 | 0.002 |  |
| PC5 _diagnosis_ | 7.900 | 2 | 747 | **<0.001** | 0.021 | SCZ, HC<BD |
| PC6 _diagnosis_ | 0.190 | 2 | 747 | 0.83 | 0.001 |  |
| PC7 _diagnosis_ | 3.228 | 2 | 747 | 0.040 | 0.009 |  |
| PC8 _diagnosis_ | 12.944 | 2 | 747 | **<0.001** | 0.033 | HC<SCZ, BD |
| Number of psychotic episodes per year, SCZ^b^ (IV) | | | | | | |
| PC1_course_ | 0.065 | 1 | 103 | 0.80 | 0.001 |  |
| PC2_course_ | 1.539 | 1 | 103 | 0.22 | 0.015 |  |
| PC3_course_ | 0.746 | 1 | 103 | 0.39 | 0.007 |  |
| PC4_course_ | 0.554 | 1 | 103 | 0.46 | 0.005 |  |
| PC5_course_ | 7.844 | 1 | 103 | **0.006** | **0.071** | upper<lower |
| PC6_course_ | 1.640 | 1 | 103 | 0.20 | 0.016 |  |
| PC7_course_ | < 0.001 | 1 | 103 | 1.0 | <0.001 |  |
| PC8_course_ | 1.639 | 1 | 103 | 0.20 | 0.016 |  |
| ^a^Total sample (SCZ + BD +HC); pairwise comparisons of estimated marginal means calculated from MANCOVA  ^b^Patient subsample (SCZ + BD); pairwise comparisons of patients in ‘upper’ and ‘lower’ quartile of number of psychotic episodes per year, and with and without lifetime comorbid substance use disorder (‘present’ and ‘absent’, respectively), were extracted from univariate test statistics of the MANCOVA  Abbreviations: Bipolar spectrum disorder (BD), Healthy controls (HC), Independent variable (IV), Multivariate analyses of covariance (MANCOVA), Principal Component (PC), Schizophrenia spectrum disorder (SCZ) | | | | | | |

Supplementary Figure 1. Principal component analysis^a^ in total sample (PC_diagnosis_) and patient subsample (PC_course_), structure matrix

^
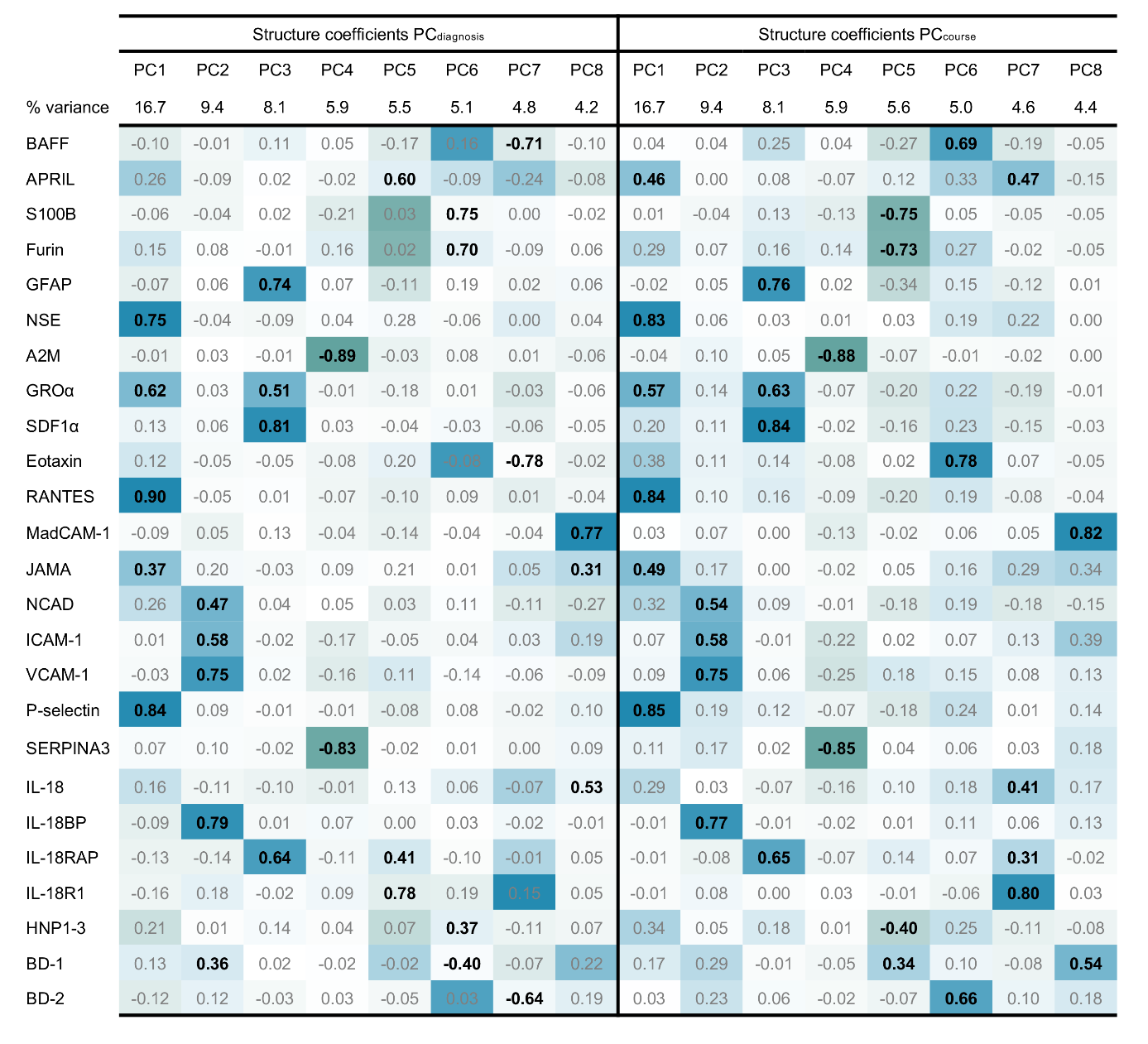
^

^a^Oblimin rotation

The structure matrix contains correlations coefficients

Abbreviations: Principal component (PC)

**References**

Andreou, D., Steen, N. E., Jørgensen, K. N., Smelror, R. E., Wedervang-Resell, K., Nerland, S., Westlye, L. T., Nærland, T., Myhre, A. M., Joa, I., Reitan, S. M. K., Vaaler, A., Morken, G., Bøen, E., Elvsåshagen, T., Boye, B., Malt, U. F., Aukrust, P., Skrede, S., Kroken, R. A., Johnsen, E., Djurovic, S., Andreassen, O. A., Ueland, T., & Agartz, I. (2021). Lower circulating neuron-specific enolase concentrations in adults and adolescents with severe mental illness. *Psychol Med*, 1-10. https://doi.org/10.1017/S0033291721003056

Engh, J. A., Ueland, T., Agartz, I., Andreou, D., Aukrust, P., Boye, B., Boen, E., Drange, O. K., Elvsashagen, T., Hope, S., Hoegh, M. C., Joa, I., Johnsen, E., Kroken, R. A., Lagerberg, T. V., Lekva, T., Malt, U. F., Melle, I., Morken, G., Naerland, T., Steen, V. M., Wedervang-Resell, K., Weibell, M. A., Westlye, L. T., Djurovic, S., Steen, N. E., & Andreassen, O. A. (2021, Sep 9). Plasma Levels of the Cytokines B Cell-Activating Factor (BAFF) and A Proliferation-Inducing Ligand (APRIL) in Schizophrenia, Bipolar, and Major Depressive Disorder: A Cross Sectional, Multisite Study. *Schizophr Bull*. https://doi.org/10.1093/schbul/sbab106

Sheikh, M. A., O`Connell, K. S., Lekva, T., Szabo, A., Akkouh, I. A., Osete, J. R., Agartz, I., Engh, J. A., Andreou, D., Boye, B., Bøen, E., Elvsåshagen, T., Hope, S., Werner, M. C. F., Joa, I., Johnsen, E., Kroken, R. A., Lagerberg, T. V., Melle, I., Drange, O. K., Morken, G., Nærland, T., Sørensen, K., Vaaler, A. E., Weibell, M. A., Westlye, L. T., Aukrust, P., Steen, V. M., Djurovic, S., Steen, N. E., Andreassen, O. A., & Ueland, T. (2022). Systemic cell-adhesion molecules (CAM) in severe mental illness-potential role of intracellular CAM-1 in linking peripheral and neuro-inflammation. *Biological Psychiatry, accepted*.

Szabo, A., O'Connell, K. S., Ueland, T., Sheikh, M. A., Agartz, I., Andreou, D., Aukrust, P., Boye, B., Boen, E., Drange, O. K., Elvsashagen, T., Engh, J. A., Hope, S., Collier Hoegh, M., Joa, I., Johnsen, E., Kroken, R. A., Vik Lagerberg, T., Lekva, T., Malt, U. F., Melle, I., Morken, G., Naerland, T., Steen, V. M., Sorensen, K., Wedervang-Resell, K., Auten Weibell, M., Westlye, L. T., Steen, N. E., Andreassen, O., & Djurovic, S. (2022, Jan). Increased circulating IL-18 levels in severe mental disorders indicate systemic inflammasome activation. *Brain Behav Immun, 99*, 299-306. https://doi.org/10.1016/j.bbi.2021.10.017
